# Uric Acid and Cognitive Function in Older Individuals

**DOI:** 10.3390/nu10080975

**Published:** 2018-07-27

**Authors:** Claudio Tana, Andrea Ticinesi, Beatrice Prati, Antonio Nouvenne, Tiziana Meschi

**Affiliations:** 1 Internal Medicine and Critical Subacute Care Unit, Geriatric-Rehabilitation Department, Azienda Ospedaliero-Universitaria di Parma, 43125 Parma, Italy; ctana@ao.pr.it (C.T.); bprati@ao.pr.it (B.P.); anouvenne@ao.pr.it (A.N.); tiziana.meschi@unipr.it (T.M.); 2 Department of Medicine and Surgery, University of Parma, 43126 Parma, Italy

**Keywords:** hyperuricemia, uric acid, dementia, cognition, Parkinson’s, Alzheimer’s

## Abstract

Hyperuricemia has been recognized as an independent cardiovascular risk factor in epidemiological studies. However, uric acid can also exert beneficial functions due to its antioxidant properties, which may be particularly relevant in the context of neurodegenerative diseases. In this paper, we critically revise the evidence on the relationship between serum uric acid levels and cognitive function in older individuals, focusing on the etiology of cognitive impairment (Alzheimer’s disease, Parkinson’s dementia, and vascular dementia) and on the interactive connections between uric acid, dementia, and diet. Despite high heterogeneity in the existing studies, due to different characteristics of studied populations and methods of cognitive dysfunction assessment, we conclude that serum uric acid may modulate cognitive function in a different way according to the etiology of dementia. Current studies indeed demonstrate that uric acid may exert neuroprotective actions in Alzheimer’s disease and Parkinson’s dementia, with hypouricemia representing a risk factor for a quicker disease progression and a possible marker of malnutrition. Conversely, high serum uric acid may negatively influence the disease course in vascular dementia. Further studies are needed to clarify the physio-pathological role of uric acid in different dementia types, and its clinical-prognostic significance.

## 1. Introduction

Hyperuricemia is defined by the presence of serum uric acid (sUA) levels above 6.8–7 mg/dL, and is associated with precipitation of monosodium urate (MSU) crystals in joints and development of gout, the most frequent inflammatory arthritis in developed countries [1]. A cut-off value of 6 mg/dL for defining hyperuricemia has been also proposed, considering that the risk of gout increases when serum uric acid levels are above this threshold [1]. sUA levels are the result of the balance between dietary purine intake, xantine oxidase activity, and renal UA excretion [2]. Recent evidence suggests that the effects of hyperuricemia go beyond the classical gouty manifestations, and it has been demonstrated that patients with hyperuricemia could have an increased cardiovascular risk (CVR), irrespective of the presence of other risk factors such as hypertension and diabetes [3]. Hyperuricemia has thus emerged as a possible independent cardiovascular risk factor. Even when serum uric acid is at high-normal level, this parameter may represent a predictor of cardiovascular adverse events. For these reasons, treating high sUA levels is pivotal for the optimal cardiovascular risk management [4].

Conversely, there is conflicting evidence about the physio-pathological role and clinical significance of sUA in influencing cognitive decline in patients with or without a specific diagnosis of dementia. The interaction between hyperuricemia and cognitive system is still debated, and results from studies are partly conflicting. Furthermore, it is also debated whether there is a different interaction between sUA and cognitive function according to the type of dementia (Alzheimer’s—AD; Parkinson’s—PD; or vascular—VD). In this literature review we report the current evidence about the relationship between UA and cognitive function, and discuss the limitations of the existing studies. We also discuss potential mechanisms that could explain this interaction, the possible role of diet, and future study directions aimed at improving the current knowledge.

## 2. Uric Acid and Dementia: Results from Large Epidemiological Studies

Several studies have investigated the interaction between UA and dementia. For this review, a literature search was performed on PubMed, Scopus, and Embase databases. The following queries were searched for the period 1999–2018: uric acid AND dementia OR cognitive impairment; uric acid AND Parkinson’s disease OR parkinsonism; uric acid AND Alzheimer’s disease; uric acid AND vascular dementia. Large studies were defined as studies which included over 500 individuals.

### 2.1. Uric Acid and Alzheimer’s Disease

Four large cohort studies have assessed the sUA–dementia relationship but their results are conflicting. In a prospective study conducted in 4618 participants ≥ 55 years old (61% women, mean age ± standard deviation (SD) 69.4 ± 8.6), Euser et al. found a dementia risk reduction in patients with higher sUA levels after adjusting for CV risk factors (Hazard Ratio (HR) 0.89 (95% confidence interval (CI) 0.80–0.99, *p* = 0.030)). Among the 1724 patients who did not develop dementia during the follow-up (mean 11.1 years), the authors observed a better cognitive performance in those with higher sUA levels (for global cognitive, executive and memory function Z-score (95% CI) 0.04 (0.00–0.07); 0.02 (−0.02 to 0.06) and 0.06 (0.02–0.11), respectively), concluding that elevated sUA levels are associated with a better cognitive performance and reduced dementia risk, independent of the CV risk [5].

Among the 814 patients (mean age 62.0 years) who underwent magnetic resonance imaging (MRI) and validated neuropsychological tests within the cross-sectional Rotterdam Scan Study, a significant association between hyperuricemia and brain white matter (WM) atrophy was found (Z-score difference: −0.07 (95% CI: −0.12; −0.01)), implying a worse cognitive performance (−0.28 (−0.48; −0.08)). These data suggest a potential role of hyperuricemia in stratifying the degree of cognitive impairment. Future studies could assess whether the variation of sUA levels could affect the MRI results over time [6].

The study by Hong and colleagues included 28,769 gouty patients with both non-vascular (including AD) and VD, and 114,742 matched controls (mean age 63.5 ± 9.7 for both groups). The patients were enrolled from the Taiwan National Health Insurance Research Database (NHIRD) and the dementia diagnosis was obtained by using the International Classification of Diseases Ninth Revision, Clinical Modification (ICD9-CM) codes. During a six-year follow-up, 1214 subjects with and 5905 subjects without gout developed dementia (102 and 542 developed AD, respectively). After age, sex, and comorbidities adjustment, the authors found a lower risk of nonvascular (including AD) dementia (HR 0.77; 95% CI: 0.72–0.83; *p* < 0.001) for those with hyperuricemia. The risk reduction of dementia associated with elevated sUA levels raises some doubts about the real need of reducing sUA in all categories of patients, highlighting also some possible beneficial function of uric acid [7].

These results were confirmed from the more recent study by Lu et al., which matched gouty and non-gouty patients also for body mass index (BMI). The authors enrolled patients from the Health Improvement Network (HIN) database, that is representative of the UK general population, and found a HR for AD among gouty patients of 0.76 (95% CI 0.66 to 0.87) at multivariate and 0.71 (95% CI 0.62 to 0.80) at univariate analysis, respectively. The indication to modulate the sUA levels in hyperuricemic patients could be suggested in patients with high BMI in order to reduce the imminent gout risk in predisposed patients, but the possible effects on cognitive function should be also considered [8].

The meta-analysis by Chen et al., which included 11 studies and 2708 participants, did not find any significant difference of sUA values between AD patients and healthy subjects (standardized mean difference (SMD) = −0.50; 95% CI: −1.23 to 0.22). However, the authors found a significant heterogeneity across individual studies (I2 = 58%, *p* < 0.01). Thus, a meta-regression analysis was performed, but the authors failed to demonstrate any significant source of heterogeneity in publication year, race, sample size, and mean age, concluding that their results had a low risk of bias after an appropriate data interpretation [9].

A more recent meta-analysis, that included 46 papers (*n* = 16,688 participants) dealing with all causes of dementia and 22 papers dealing with AD diagnosis, found lower sUA values in patients with a diagnosis of dementia (SMD −0.33 (95% CI), *p* < 0.001), with a stronger association between sUA and AD, as compared to PD patients (SMD −0.33 (95% CI) *p* < 0.001, and −0.67 (95% CI) *p* < 0.001, respectively). These data could suggest a neuroprotective role of UA on cognitive function, showing its best influence on patients with the AD rather than other dementia types. However, no correlation was found between the Mini-Mental State Examination (MMSE) test score and sUA levels (*r* = 0.08, *p* = 0.27), except for the subset of patients with PD (*r* = 0.16, *p* = 0.003) [10].

The inverse association between sUA and AD risk was confirmed also in the meta-analysis by Du et al. The authors identified 21 case-control and 3 cohort studies (total subjects included: 10,953). AD patients showed lower sUA levels than controls (weighted mean difference (WMD) = −0.77 mg/dl, 95% CI −2.28 to −0.36, *p* = 0.0002), and higher sUA were associated with a significant reduced AD risk (RR 0.66, 95% CI 0.52–0.85, *p* = 0.001), confirming the hypothesis of a neuroprotective role of sUA [11]

### 2.2. Uric Acid and Parkinson’s Dementia

Although earlier studies have found no significant difference in terms of sUA levels between PD patients and healthy subjects [12,13], a neuroprotective role of UA was found later in different studies. The first meta-analysis including six studies with a total of 33,185 subjects found a 33% reduction of PD incidence in patients with higher sUA levels (RR = 0.67; 95% CI, 0.50–0.91). A subgroup analysis confirmed the significant neuroprotective role of sUA, possibly through its antioxidant effects, only in males (RR = 0.60; 95% CI, 0.40–0.90) [14].

Accordingly, a recent meta-analysis of seven studies found lower sUA values in PD patients than controls (SMD −0.67, 95% CI, *p* = 0.001), and the authors documented a positive correlation between sUA levels and MMSE (*r* = 0.155; *p* = 0.003). The authors did not find any significant publication bias (*p* = 0.326 using Egger’s plots), and these data seem to confirm the beneficial effects that sUA could have in PD patients, in term of improvement of cognitive function. Traditionally, PD has been considered as an irreversible disorder. However, these results suggest a positive influence on the neurodegeneration process of the disease, and some reversible effects could be obtained by modulating sUA levels [10].

A case-control study, enrolling 90,214 participants from 3 cohorts, identified 388 patients with PD (202 M and 186 F), and demonstrated an inverse relationship between sUA and PD onset only in males (*p* = 0.049), and not in females (*p* = 0.44). The finding that higher sUA levels are associated with a reduced PD risk can confirm the neuroprotective role of urate in men and its potential action in slowing the progression of disease [15].

More recently, the meta-analysis of Wen et al. which investigated 4646 participants (2379 PD patients and 2267 controls) from 13 studies, confirmed that PD patients have lower sUA levels than controls (SMD: −0.49, 95% CI: (−0.67, −0.30), Z = 5.20, *p* < 0.001), without any significant difference in term of age, sex, and geographic provenience. The patients with middle–late disease and more disabling symptoms, as evaluated with higher Hoehn and Yahr (H&Y) scale, had significantly lower sUA values than those of earlier stages of disease with lower H&Y scale (SMD = 0.63, 95% CI (0.36, 0.89), z = 4.64, *p* < 0.001), suggesting a linear correlation between sUA levels and global cognitive and functional decline. sUA could thus have a key role in predicting the disease progression and clinical worsening [16].

Furthermore, recent data have underlined that high sUA levels may be associated with lower non-motor symptoms such as fatigue in the early stages of PD (OR = 0.693; *p* = 0.0408). These results could be related to the antioxidant effects that UA may exert in PD patients [17], and some authors hypothesize that the modulation of serum levels could be useful to prevent the manifestations of the disease [18].

### 2.3. Uric Acid and Vascular or Mixed Dementia

Study findings about the relation between UA and VD are controversial. The study by Hong et al. found a lower risk of developing VD in gouty patients, with 210 new cases as compared to the 991 in patients without gout at a mean follow-up of 4.5 ± 2.0 and 4.4 ± 2.0, respectively (HR: 0.76; 95% CI: 0.65–0.88; *p* < 0.001). The authors obtained the diagnosis of dementia by using ICD9-CM codes and matched 28,769 patients for age and sex with 114,742 controls [7].

The meta-analysis by Khan et al., however, did not find a significant association between sUA and vascular or mixed dementia (SDM −0.05 (95% CI) and (SDM 0.19 (95% CI), respectively) and no correlation was found between sUA levels and MMSE scores. Heterogeneity among different studies, the case-control design of many of them and the absence of longitudinal data with clinical follow-up were the most relevant limitations and the main sources of possible biases [10].

Recently, a population-based cohort study, including 1598 subjects (mean age 72.4 ± 4.1 years, 38.3% male) found an incidence rate of dementia of 8.2/1000 person-years (100 new cases) during a 10.1-year follow-up period. The authors found a significant risk of vascular or mixed dementia in patients with higher sUA levels (HR = 3.66, 95% CI 1.29 to 10.41, *p* = 0.015), while sUA did not predict the onset of AD (HR = 1.55 (95% CI 0.92 to 2.61), *p* = 0.10). Stroke occurrence during the follow-up, and a stroke-related cognitive decline, could have influenced this risk, but these results demonstrate a risk difference as compared with the other forms of dementia. It is reasonable to hypothesize a specific action of sUA in term of damage on the vascular system acting differently from the traditional antioxidant effects that occur in the neurodegenerative forms of dementia. A thorough clinical evaluation and risk factor profiling should be addressed in order to identify the patients at highest risk of stroke and vascular-related cognitive impairment, that could be partly influenced by elevated sUA levels [19].

### 2.4. Uric Acid and Mild Cognitive Impairment

Interestingly, the relationship between sUA and cognitive function seems to be emphasized in the early stages of cognitive dysfunction, and not only in advanced dementia. Previous studies have demonstrated a protective role of UA against the progression of cognitive impairment [5,20,21]. Although a subsequent meta-analysis did not find a significant percent change of UA in plasma/serum of patients with MCI compared with subjects with normal cognitive function [22], recent studies support the theory about a neuroprotective role of UA [23]. Namely, significantly lower sUA levels were found in 58 patients affected by MCI than in 57 controls ((292.28 ± 63.71 μmol/L vs. 322.49 ± 78.70 μmol/L, respectively; *p* < 0.05). Despite the limited sample size, this study demonstrated a significant positive correlation between the MMSE scores (both for each point and the total score), and sUA levels (*p* < 0.05), supporting a mild, but significant, protective role of UA against the onset of MCI at a multivariate logistic regression analysis (odds ratio OR = 0.999, 95% CI = 0.987–0.999). The authors, in line with the hypothesis by Yu et al. [18] concluded that a modulation of sUA levels could be useful to prevent the MCI incidence [24].

These data were confirmed in a large cross-sectional study conducted in a Chinese community of 2102 elderly (mean age 71.2 ± 6.6 years old, 59.7% F) between 2009 and 2010. The authors found a linear decrease of MCI prevalence with increasing sUA values, and ORs for MCI was 1.65 (95% CI: 1.12–2.43) and 1.92 (95% CI: 1.02–3.35) for the highest quarters in men and women, respectively [25]. According to these data, the neuroprotective effects of sUA could start a long time before the onset of clinical manifestations, suggesting a dual benefit in term of risk reduction and clinical improvement of overt disease.

Table 1 shows the cohort studies which assessed the relationship between UA and risk of dementia.

## 3. Mechanisms Underlying the Relation between UA and Cognitive Function

### 3.1. Oxidative Stress

The mechanisms by which UA can influence the cognitive function are still unknown. Oxidative stress seems to play a key role in the pathogenesis of the most of causes of dementia, in particular AD and PD [26,27,28,29,30]. A meta-analysis found a significant reduction of total antioxidant capacity (16%, Effect size Hedge’s g −0.85 (CI −1.29 to −0.41)) and sUA levels (25%, −0.59 (CI −1.26 to −0.09)) in AD patients than controls [22]. These data were recently confirmed along with a reduction trend of sUA and other serum antioxidants—such as α- and β-carotene; lycopene; lutein; and vitamins A, C, and E—that could contribute to increase the oxidative stress in these patients [31].

In PD patients, oxidative stress due for example to a chronic exposure to environmental toxins, low antioxidant levels in the brain or of free radical scavenging enzymes, mitochondrial dysfunction seems one of the leading causes of neurodegeneration and can interact with multiple neuronal circuits and pathways [32,33,34].

Several studies have reported antioxidant effects of sUA, similar to those of ascorbate, another important antioxidant in humans. UA acts as a direct scavenger of oxygen and hydroperoxyl radicals, singlet oxygen, oxo-heme oxidants and can make stable complexes with iron ions [35,36,37]. Therefore, lower sUA levels can be associated with a reduced protection towards oxidative stress and can increase the risk of cognitive dysfunction in these patients. Some authors found a linear correlation between sUA levels and UA from cerebrospinal fluid (CSF, *r* = 0.669, *p* = 0.001), and also an association between the impairment of blood–brain barrier with higher UA levels in CSF (*p* = 0.028), supporting the hypothesis of a strong influence of UA on brain and cognitive system [38]. Interestingly, the inverse association between UA levels and AD and PD was confirmed also from cortical and striatal tissues in a post-mortem analysis, supporting the theory of a neuroprotective action that UA could have on cognitive system [39].

However, some authors have also suggested that UA can even have pro-oxidant effects, and this dual action may partly depend on its chemical microenvironment. In fact, UA can both reduce the oxidation of native LDLs and increase the oxidation of LDL particles which are already oxidized, depending on the presence of transition metals [40,41].

### 3.2. UA and Interaction with β-Amyloid

It has been demonstrated also that the neurotoxic influence of UA could be exerted by amplifying the β-amyloid effects involved in AD. In experimental models, some authors found that the cell incubation with a 40 µM dose of UA reduced significantly the cell viability, increased the amyloid-induced proapoptotic effect and the βPPARβ/δ expression [42].

By contrast, some authors found that higher UA levels reduced the detrimental effects of Amyloid β1-42, a CSF biomarker on cognitive function, and documented an improvement of MMSE and the AD Assessment Scale-Cognitive Subscale (ADAS-cog) in a follow-up period of 2.9 years [43].

Considered globally, these data reveal that there is a strong relationship between UA and Aβ amyloid. However, the precise role of uric acid in modulating amyloid deposition and histological aspects of AD is far from being fully understood. These aspects should be prospectively investigated because it could be a different interaction, perhaps related to a time-dependent neuroprotective effect of sUA that can influence differently the cognitive risk impairment in these subjects.

### 3.3. Inflammation, Endothelial Dysfunction, and Vascular Damage

Another point of connection between UA and cognitive system, in particular in patients with VD, could be represented by vascular damage. It has been documented that elevated sUA levels can be associated with increased inflammatory response to oxidative stress, endothelial dysfunction, vascular damage and remodeling, that could explain the increased risk of VD in some patients [44,45,46,47].

Inflammation can be an important link between high sUA values and risk of VD. Some studies found indeed a positive correlation between higher C-reactive protein (CRP), IL-6 levels and sUA levels [19,48] and between inflammatory markers and white matter hyper-intensity (WMH, (*p* < 0.01)), lower gray matter (*p* = 0.001) and hippocampal (*p* = 0.01) volumes, that are indirect markers of brain atrophy, supporting the hypothesis of a strong connection between elevated sUA levels, inflammation, and VD [49]. However, the direct link between hyperuricemia, inflammation, and cognitive dysfunction has been demonstrated only recently. Some authors found that a reduction of UA-related hippocampal inflammation, obtained by inhibiting the nuclear factor (NF)-κB signaling pathways, is associated with improvement of cognitive function in rats, and that knock-out mice are protected against cognitive impairment. The authors have also found a significant increase of hippocampal gliosis related to sUA levels both in humans and rats, but they did not address the correlation between inflammation and cognitive dysfunction in humans [50].

### 3.4. Diet, Uric Acid, and Dementia

Diet plays a central role in the onset of dementia, even if not completely understood to date. In their recent extensive systematic review, Solfrizzi et al. showed that dietary patterns and synergistic actions of nutrients, rather than single nutrients or foods, determine the risk of developing dementia and the risk of a faster clinical course towards end-stage dementia, once the disease has established. The adherence to Mediterranean-style or Dietary Approach to Stop Hypertension (DASH)-style diets is significantly associated with a reduced risk of dementia and dementia progression [51].

Interestingly, both these dietary patterns share high fruit and vegetable intakes, and are associated with reduced sUA levels and reduced risk of hyperuricemia in both adult and older subjects [51,52]. This scenario apparently contradicts the assumption that uric acid exerts a protective effect against dementia.

However, the relationship between diet, uric acid, and cognitive function may be very difficult to understand and explain. Hyperuricemia has been classically associated with the intake of purine-rich foods, that are generally abundant in high-protein diets. The review by Solfrizzi et al. highlighted that the intake of high-protein foods is not associated with the risk of dementia, but instead showed that saturated fatty acids—i.e., fats of animal origin—are correlated with dementia and its progression [52,53]. On the other side, some studies performed in populations of Asian origin have underlined that the risk of hyperuricemia seems to be unrelated with the dietary intake of meat, being instead dependent on low fruit and vegetable consumption, high seafood intake, and unhealthy dietary patterns [54,55,56].

Thus, uric acid may represent a complex mediator of dietary patterns, rather than of a single nutrient intake, influencing cognitive function. Uric acid may be at the cross-road between diet and dementia physio-pathology, and, in some cases, may simply represent a metabolic marker of complex phenomena rather than an active mediator determining disease progression. Interestingly, the results from the Prospective Investigation of the Vasculature in Uppsala Seniors (PIVUS), a population-based study performed on 850 Swedish subjects aged 70 years old, suggested that uric acid is not an independent mediator of endothelial function, being instead influenced by body composition and hormonal milieu [57]. The influence of uric acid on cognitive function should also be interpreted in the light of these concepts.

Moreover, in older patients with MCI or dementia, malnutrition is frequently an issue, implying long-term adverse outcomes [58]. The so-called “anorexia of aging”, due to the age-related rearrangements of the gastrointestinal system and hormonal regulation of appetite, is the physio-pathological mechanism frequently leading to malnutrition [59]. Low sUA is generally associated with this condition. Hypouricemia is a well-established marker of poor nutritional status, used especially in the context of patients undergoing chronic maintenance hemodialysis [60,61]. As such, the proposed relationship between low sUA and poorer cognitive function, supported by most of the above-mentioned studies, may reflect the presence of malnutrition.

Dementia, especially in its advanced stages, is a well-recognized risk factor for malnutrition. However, there are also some data indicating that the presence of malnutrition in older individuals may influence the onset of cognitive impairment and dementia, and accelerate its clinical course [62,63]. Future studies investigating the relationship between uric acid and cognitive function should also incorporate nutritional screening and evaluation of the presence of malnutrition in their design so that they may better elucidate whether the possible inverse relationship between sUA and cognitive function in the elderly is mediated by the presence of malnutrition.

### 3.5. Genetic Syndromes and Cognitive Decline

The close relationship between sUA and cognitive function could be suggested also by the association between some genetic abnormalities of the purine metabolism and neurological impairment. Lesch–Nyhan Syndrome is a rare X-linked disease characterized by hyperuricemia, gout, dystonia, microcephaly, mental retardation, and self-injurious behavior, and is caused by the hypoxanthine-guanine phosphoribosyltransferase (HPRT1) enzyme deficiency and altered purine metabolism. Although first hypothesis linked uric acid overproduction and neurological symptoms, it is actually unknown why some disease variants (with >1% of enzymatic activity) do not manifest with neurological impairment and self-mutilation. Furthermore, while allopurinol treatment can successfully manage gout and liver complications of the disease, it does not change the neurological course, supporting the theory that there are also other mechanisms that could influence the brain disease in these patients [64].

Primary renal hypouricemia (PRH) is another rare condition characterized by increased UA excretion from a reduced reabsorption due to *SLC22A12* or *LC2A9* gene defects, that code for the *hURAT1* and *GLUT9* transporters, respectively. PRH is associated with low sUA levels, nephrolithiasis, and exercise-induced acute failure (EIARF). Several authors have found an association between some neurological alterations of the posterior reversible encephalopathy syndrome and EIARF, supporting the hypothesis of a relation between changes of sUA levels and altered cognitive function [65,66,67].

Although these diseases would suggest the presence of an inherited predisposition to cognitive dysfunction in patients with congenital disorders of uric acid, there is lack of data on the specific influence of gene variants on the neurologic manifestations, and on the specific contribution of UA.

**Figure 1:**
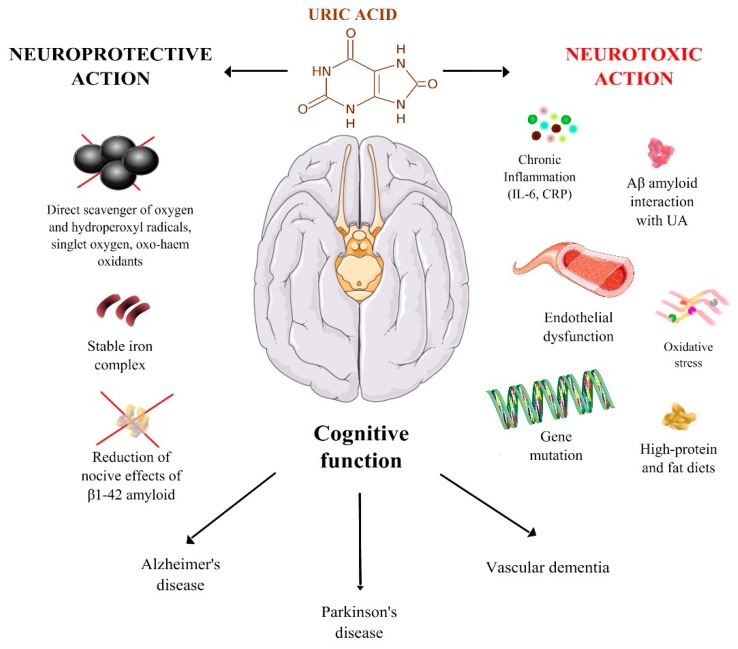
Summary of the main mechanisms of interaction between uric acid and cognition in the physio-pathology of different types of dementia. The specific mechanisms are discussed in the text.

Figure 1 summarizes the main mechanisms of interaction between UA and cognitive system.

## 4. Study Limitations at the Basis of Conflicting Evidence

There are some limits that could partly explain the conflicting evidence about the relation between UA and cognitive function, in particular about the contrasting neuroprotective or neurotoxic action of UA.

Several meta-analyses report a significant study heterogeneity. Furthermore, most of studies are case control or cross-sectional and analyze the immediate relationship between UA and cognitive function, without a specific follow-up over time [68]. More information could be obtained with population-based studies with prospective design aimed at evaluating patients free from dementia at baseline, because sUA levels can influence differently the cognitive function over time, where a neuroprotection trend is observed at a longer follow-up from baseline [10].

Another limitation emerging from studies is the circumstance that the diagnosis of dementia is often assessed by using *ICD9-CM* codes and is not based on standardized clinical and imaging (e.g., MRI) criteria. *ICD9-CM* codes are often limited by the clinician experience, and sometimes only a generic definition of dementia or cognitive decline is given, without a specific description of the dementia subtype. This is a possible, very important source of bias, considering that dementia often goes undiagnosed when patients are evaluated outside a geriatric or neurologic specialty context, especially in the oldest old [69]. Few studies have investigated the cognitive function with standardized scales (e.g., MMSE), and there is little information about the potential influence of comorbidities (e.g., metabolic syndrome, hypertension, renal failure) on sUA and therefore on cognitive function [70,71]. Considering the complex and strong interaction between cognitive function, physical status, and comorbidities [72], it could be interesting to explore the specific influence of a nutritional marker such as UA in patients with different forms of dementia.

Actually, the ongoing COPPADIS-2015 (COhort of Patients with PArkinson’s DIsease in Spain, 2015) study is evaluating global aspects of PD patients, including comorbidity, biomarkers, and their influence on cognitive system. Study completion is estimated around May 2022 and will provide important information on the natural history and specific aspects of this disease [73].

## 5. Conclusions

The relationship between sUA and cognitive system remains a debated issue. Although the present evidence supports the hypothesis of a neuroprotective action of UA towards cognitive system in AD and PD patients, and toxic effects in those with VD, several studies raise some concerns about the specific influence. Most causes of conflict between results are represented by study heterogeneity and low number of perspective studies. Future studies should be aimed at assessing the variation of sUA levels (and its correlation with brain function) over time, because most relevant effects are observed longitudinally. Other study directions could include the evaluation of the effects of sUA-lowering therapies and the potential influence of comorbidities on the cognitive system.

## Figures and Tables

**Table 1:** Large studies assessing the relationship between UA and risk of dementia

First Author, Journal, Year [Ref]	Type of Study	Population	Duration	Dementia Subtype	Main Results
Euser et al. 2009 [5]	Prospective	7983 subjects	11 years	AD	Dementia risk reduction with higher sUA levels (HR 0.89; 95% CI: 0.80–0.99, *p* 0.030).
Verhaaren et al. 2013 [6]	Cross-sectional analysis from the Rotterdam study	814 persons of the Rotterdam Study	N/A	AD	Significant relation between hyperuricemia and worse cognitive performance (Z score difference −0.28 (−0.48; −0.08)).
Hong et al. 2015 [7]	Prospective	28,769 gouty patients and 114,742 controls	6 years	AD and VD	Lower risk of AD (HR 0.77; 95% CI: 0.72–0.83; *p* < 0.001) and of VD (HR: 0.76; 95% CI: 0.65–0.88; *p* < 0.001) in patients with hyperuricemia
Lu et al. 2016 [8]	Prospective	59,224 gouty patients and 1942 controls	5 years	AD	Higher risk of dementia in gouty patients (HR of 0.76 (95% CI 0.66 to 0.87) at multivariate and 0.71 (95% CI 0.62 to 0.80) at univariate analysis).
Gao et al. 2016 [15]	Prospective	90,214 subjects	34 years	PD	Lower risk (0.63 (95% CI 0.35, 1.10; *p* = 0.049)) of developing PD in men with higher sUA levels
Latourte et al. 2018 [19]	Prospective	1598 subjects	10.1 years	VD	Significant risk of VD or mixed dementia in patients with higher sUA levels (HR = 3.66, 95% CI: 1.29–10.41, *p* = 0.015)
Liu et al. 2017 [25]	Prospective	2012 subjects	2 years	MCI	Risk reduction of MCI with increasing sUA values (OR 1.65(95% CI: 1.12–2.43) and 1.92 (95% CI: 1.02–3.35) for the highest quarters in men and women, respectively).

sUA = serum uric acid; AD = Alzheimer’s dementia; PD = Parkinson’s dementia; VD = vascular dementia; MCI = mild cognitive impairment; HR = hazard ratio; OR = odds ratio; CI = confidence interval.
